# ﻿Morpho-phylogenetic evidence reveals novel species and new records of *Nigrograna* (Nigrogranaceae) associated with medicinal plants in Southwestern China

**DOI:** 10.3897/mycokeys.110.132628

**Published:** 2024-10-23

**Authors:** Hong-Zhi Du, Yu-Hang Lu, Ratchadawan Cheewangkoon, Jian-Kui Liu

**Affiliations:** 1 School of Life Science and Technology, Center for Informational Biology, University of Electronic Science and Technology of China, Chengdu 611731, Sichuan Province, China; 2 School of Pharmacy, Guizhou University of Traditional Chinese Medicine, Guiyang 550025, Guizhou Province, China; 3 Department of Entomology and Plant Pathology, Faculty of Agriculture, Chiang Mai University, Chiang Mai 50200, Thailand; 4 Innovative Agriculture Research Centre, Faculty of Agriculture, Chiang Mai University, Chiang Mai 50200, Thailand

**Keywords:** 4 new taxa, Dothideomycetes, multi-locus, phylogeny, sexual morph, taxonomy

## Abstract

During a survey of saprobic fungal niches in Southwestern China, eighteen ascomycetous collections of *Nigrograna* (Nigrogranaceae, Pleosporales, Dothideomycetes) were found on dead branches of medicinal plants. These taxa were characterized and identified based on morphological and culture characteristics, and phylogenetic analyses of a combined the internal transcribed spacer region of rDNA (ITS), nuclear large subunit rDNA (28S, LSU), RNA polymerase second-largest subunit (rpb2), nuclear small subunit rDNA (18S, SSU), and translation elongation factor 1-alpha (*tef1-α*) sequence dataset also confirmed their placement. As a result, four novel species, namely *Nigrogranacamelliae*, *N.guttulata*, *N.longiorostiolata* and *N.neriicola* were described. Additionally, four new host records of *N.acericola*, *N.magnoliae*, *N.oleae* and *N.thymi* were introduced. Furthermore, this study addresses the taxonomic status of *N.trachycarpi*, proposing its synonymy under *N.oleae*. Detailed illustrations, descriptions and informative notes for each newly identified taxon and novel host record are provided in this study.

## ﻿Introduction

The utilization of medicinal plants is integral to disease prevention and treatment in human life (Nalawade et al. 2003; Cole et al. 2007; Schmidt 2017; Rahman et al. 2019). These plants harbor diverse biological compounds that hold potential for drug development due to their rich reservoir of bioactive ingredients (Samy and Gopalakrishnakone 2007; Ali et al. 2021; Atanasov et al. 2021). Southwestern China, recognized as one of the primary regions for traditional Chinese herbal medicine, boasts remarkable diversity in medicinal plant species. This diversity is largely driven by the region’s unique karst landforms, which promote species differentiation and abundance (Huang et al. 2012; Taylor et al. 2014; Lu et al. 2018; Guo et al. 2020; Shan et al. 2022). Recent studies in Southwest China have revealed novel micro-fungi species associated with medicinal plants, including pathogens (Abtahi and Nourani 2017), saprobes (Du et al. 2022a; Sun et al. 2023; Wu et al. 2024) and endophytes (Helaly et al. 2018; Keshri et al. 2021; Du et al. 2022b), highlighting the potential of these plants as reservoirs for discovering fungal diversity.

Nigrogranaceae (Pleosporales, Dothideomycetes) was established by Jaklitsch and Voglmayr (2016) based on morpho-molecular evidence, with *Nigrograna* as the type genus. The divergence of Nigrogranaceae is established at approximately 79 (44–124) Mya in crown age and 131 (86–180) Mya in stem age (Liu et al. 2017). Initially, the genus *Nigrograna* was proposed by de Gruyter et al. (2012) to accommodate *Pyrenochaetamackinnonii* (a pathogenic species in humans isolated from a mycetoma patient), which was later synonymized as *Nigrogranamackinnonii*. However, phylogenetic studies revealed that *N.mackinnonii* was closely related to *Biatriosporamarina*, the type species of the monotypic genus *Biatriospora* (Ahmed et al. 2014), leading to the reclassification of *N.mackinnonii* as *Biatriosporamackinnonii* (Ahmed et al. 2014). Jaklitsch and Voglmayr (2016) subsequently proposed the family Nigrogranaceae, describing three new taxa that differed significantly from *Biatriospora* in morphology and ecology. Hongsanan et al. (2020) further revised the taxonomic status of *Biatriospora* and *Nigrograna*, suggesting that both genera should be retained. To date, there are 37 species epithets of *Nigrograna* listed in Index Fungorum (http://www.indexfungorum.org/Names/Names.asp; accessed 7 September 2024).

Most members of *Nigrograna* have cryptic morphological characters, leading Jaklitsch and Voglmayr (2016) to classify them as cryptic species. The sexual morphs of *Nigrograna* are characterized by having globose to subglobose ascomata with ostiole, multi-layered peridium, clavate and fissitunicate asci, fusoid to narrowly ellipsoid, straight or curved, septate, and smooth or verruculose ascospores (Jaklitsch and Voglmayr 2016; Zhang et al. 2020; Lu et al. 2022). In contrast, the asexual morphs are defined by globose to subglobose or pyriform pycnidia, filiform and branched conidiophores, hyaline, phialidic and discrete conidiogenous cells, sub-hyaline, aseptate and ellipsoidal conidia (de Gruyter et al. 2012; Jaklitsch and Voglmayr 2016; Lu et al. 2022). The life modes of *Nigrograna* species are diverse, ranging from endophytic and saprobic to pathogenic (in human) (Kolařík 2018; Zhao et al. 2018; Lu et al. 2022; Li et al. 2023). These species have been reported from various hosts in terrestrial, marine, and freshwater habitats (Hyde et al. 2017; Tibpromma et al. 2017; Dayarathne et al. 2020; Lu et al. 2022; Bundhun et al. 2023; Hu et al. 2023; Li et al. 2023; Senanayake et al. 2023; Shu et al. 2023), underscoring the broad ecological diversity of this genus. In recent years, an increasing number of new species and records of *Nigrograna* have been reported from various hosts in China. Most of these species have been identified as saprotrophic fungi from terrestrial habitats (Tibpromma et al. 2017; Boonmee et al. 2021; de Silva et al. 2022; Lu et al. 2022; Hu et al. 2023; Li et al. 2023; Liu et al. 2024; Ren et al. 2024; Xu et al. 2024). However, reports of *Nigrograna* occurring on medicinal plants are limited. Given the ecological and economic importance of these plants, it is essential to explore the taxonomy and phylogeny of *Nigrograna* species associated with medicinal flora. Such investigations will deepen our understanding of fungal diversity in these specialized niches and may reveal new insights into the potential applications of these fungi.

This study focuses on elucidating the diversity of Nigrogranaceae in Southwestern China, identifying eight species associated with medicinal plants. We aim to describe these novel findings and contribute to the understanding of fungal diversity in this region. Through a combination of morphological comparisons and multi-locus phylogenetic analyses, we introduce four new species and four new host records, supported by both morphological and phylogenetic evidence.

## ﻿Materials and methods

### ﻿Collection and examination of specimens

Specimens in this study were collected from medicinal plants of nine families (Apocynaceae, Berberidaceae, Buxaceae, Celastraceae, Eucommiaceae, Fabaceae, Primulaceae, Rutaceae and Theaceae) in Southwest China during 2021 and 2023, viz., (1) Guizhou Province (26°30'43"N−26°32'18′′N, 106°39'32"E−106°41'48′′E, elevation 1,127–1,155 m); (2) Sichuan Province (29°29'1"N−31°8'4"N, 103°2'23"E−104°14'19"E, elevation 504–1,200 m); (3) Yunnan Province (21°55'53′′N−25°14'27′′N, 101°23'19′′E−102°44'28′′E, elevation 505–1,922 m). The sampling information (date, host, place, GPS, etc.) was recorded. Samples were packaged in envelopes and brought to the laboratory following the method described by Senanayake et al. (2020). Morphological observations were made using a Motic SMZ (Stereoscopic Zoom Microscope) 168 Series dissecting microscope (Motic, Xiamen, China) for fungal structures on a natural substrate. Fruiting bodies were collected using a syringe needle and transferred to a drop of tap water on a clean slide. The features were examined and photographed using a Nikon ECLIPSE Ni-U compound microscope fitted with a Nikon DS-Ri2 digital camera. Measurements were made with the Tarosoft Image Frame Work v. 0.9.7 software following the procedures outlined by Liu et al. (2010), and images used for photo plates were processed with Adobe Photoshop CC 2018 software (Adobe Systems, San Jose, CA, USA). Single spore isolations were made on potato dextrose agar (PDA, Oxoid) or water agar (WA, Oxoid) and later transferred onto new PDA plates following the methods described in Senanayake et al. (2020). Incubation and cultural growth were observed at 25 °C in dark and pure cultures were obtained.

Herbarium specimens were deposited in the Herbarium of Cryptogams, Kunming Institute of Botany Academia Sinica (HKAS), Kunming, China, and the herbarium of University of Electronic Science and Technology (HUEST), Chengdu, China. The pure cultures obtained in this study were deposited in the China General Microbiological Culture Collection Center (CGMCC) in Beijing, China and the University of Electronic Science and Technology Culture Collection (UESTCC), Chengdu, China. Names of the new taxa were registered in MycoBank (http://www.mycobank.org/).

### ﻿DNA extraction, PCR amplification and sequencing

Isolates were grown in PDA medium at 25 °C in dark for three weeks to one month. Fungal mycelia were scraped off and transferred to 1.5 mL microcentrifuge tubes using a sterilized lancet for genomic DNA extraction. Fungal DNA was extracted from mycelia (about 50–100 mg) using the Trelief TM Plant Genomic DNA Kit (TsingKe Co., Beijing, China). Five different gene regions were amplified by Polymerase Chain Reaction (PCR). The internal transcribed spacer region of rDNA (ITS), nuclear large subunit rDNA (28S, LSU), nuclear small subunit rDNA (18S, SSU), RNA polymerase second-largest subunit (rpb2) and translation elongation factor 1-alpha (tef1-α) were selected for the study. The primers used were LR0R/LR5 for LSU (Vilgalys and Hester 1990), NS1/NS4 for SSU (White et al. 1990), ITS5/ITS4 for ITS (White et al. 1990), fRPB2-5F and fRPB2-7cR for *rpb2* (Liu et al. 1999) and TEF1-983F/TEF1-2218R for *tef1-α* (Rehner and Buckley 2005). Amplifications were performed in a 25 µL reaction volume containing 9.5 µL of ddH_2_O, 12.5 µL of 2× Taq PCR Master Mix with blue dye (Sangon Biotech, Shanghai, China), 1 µL of DNA template and 1 µL of each primer. The amplification condition for ITS, LSU, SSU, and *tef1-α* consisted of initial denaturation at 94 °C for 3 min, followed by 40 cycles of 45 s at 94 °C, 50 s at 55 °C and 1 min at 72 °C, and a final extension period of 10 min at 72 °C. The amplification condition for the *rpb2* gene consisted of initial denaturation at 95 °C for 5 min; followed by 37 cycles of 15 s at 95 °C, 50 s at 56 °C and 2 min at 72 °C, and a final extension period of 10 min at 72 °C. The PCR product purification and sequencing were performed at Beijing Tsingke Biotechnology (Chengdu) Co., Ltd., Chengdu, China.

### ﻿Phylogenetic analyses

In this study, the taxa included in the phylogenetic analyses were selected and obtained from previous studies and GenBank (Table 1), with a total of 67 taxa. *Occultibambusapustula* (MFLUCC 11-0502) and *O.bambusae* (MFLUCC 13-0855) (Occultibambusaceae, Pleosporales) were selected as outgroup taxa. Single-locus alignments were made in MAFFT v. 7 (http://mafft.cbrc.jp/alignment/server/) (Katoh and Standley 2013) and checked visually using AliView (Larsson 2014). The alignments were trimmed using trimAl v 1.2 (Capella-Gutierrez et al. 2009). Five single-locus alignments were combined using SequenceMatrix 1.7.8 (Vaidya et al. 2011). Maximum likelihood (ML) and Bayesian inference (BI) analyses were employed to assess phylogenetic relationships as detailed in Dissanayake et al. (2020).

**Table 1.** Taxa used in the phylogenetic analyses and the corresponding GenBank accession numbers.

Taxa names	Strain/Specimen number	GenBank accession numbers					References
		ITS	LSU	*rpb2*	SSU	*tef1-α*	
*Nigrogranaacericola*	CGMCC 3.24957 ^T^	OR253153	OR253312	N/A	N/A	OR263572	Li et al. (2023)
***Nigrogranaacericola***	**UESTCC 23.0208**	**PP812425**	**PP812460**	**PP838917**	**PP812443**	**PP838935**	**In this study**
***Nigrogranaacericola***	**UESTCC 23.0191**	**PP812426**	**PP812461**	**PP838918**	**PP812444**	**PP838936**	**In this study**
*Nigrogranaantibiotica*	CCF 4378 ^T^	JX570932	KF925327	N/A	KF925328	JX570934	Kolařík (2018)
*Nigrogranaantibiotica*	CCF 4498	LT221894	LT221895	N/A	N/A	N/A	Kolařík (2018)
*Nigrogranaaquatica*	MFLUCC 17-2318 ^T^	MT627705	MN913705	N/A	N/A	N/A	Dong et al. (2020)
*Nigrogranaasexualis*	ZHKUCC 22-0214 ^T^	OP450965	OP450971	OP432241	OP450979	OP432245	Lu et al. (2022)
***Nigrogranacamelliae***	**CGMCC 3.25625 ^T^**	**PP812431**	**PP812466**	**PP838923**	**PP812449**	**PP838939**	**In this study**
***Nigrogranacamelliae***	**UESTCC 23.0197**	**PP812432**	**PP812468**	**PP838924**	**PP812450**	**PP838940**	**In this study**
*Nigrogranacangshanensis*	MFLUCC 15-0253 ^T^	KY511063	KY511064	N/A	KY511065	N/A	Tibpromma et al. (2017)
*Nigrogranacarollii*	CCF 4484 ^T^	LN626657	LN626682	LN626662	LN626674	LN626668	Kolařík (2018)
*Nigrogranachromolaenae*	MFLUCC 17-1437 ^T^	MT214379	MT214473	N/A	N/A	MT235801	Mapook et al. (2020)
*Nigrogranacoffeae*	ZHKUCC 22-0210 ^T^	OP450967	OP450973	OP432243	OP450981	OP432247	Lu et al. (2022)
*Nigrogranacoffeae*	ZHKUCC 22-0211	OP450968	OP450974	OP432244	OP450982	OP432248	Lu et al. (2022)
*Nigrogranafuscidula*	CBS 141556 ^T^	KX650550	N/A	N/A	N/A	KX650525	Jaklitsch and Voglmayr (2016)
*Nigrogranafuscidula*	CBS 141476	KX650547	N/A	KX650576	KX650509	KX650522	Jaklitsch and Voglmayr (2016)
*Nigrogranaguizhouensis*	CGMCC 3.25501 ^T^	OR680498	OR680565	OR842915	OR680867	OR858897	Zhang et al. (2024)
*Nigrogranaguizhouensis*	ZY22.020	OR680499	OR680566	OR842916	OR680868	OR858898	Zhang et al. (2024)
***Nigrogranaguttulata***	**CGMCC 3.25689 ^T^**	**PP812433**	**PP812469**	**PP838925**	**PP812451**	**PP838941**	**In this study**
***Nigrogranaguttulata***	**UESTCC 23.0295**	**PP812434**	**PP812470**	**PP838926**	**PP812452**	**PP838942**	**In this study**
*Nigrogranaheveae*	ZHKUCC 22-0284 ^T^	OP584490	OP584488	OP750374	OP584492	OP750372	Hyde et al. (2023)
*Nigrogranahydei*	GZCC 19-0050 ^T^	MN387225	MN387227	N/A	N/A	MN389249	Zhang et al. (2020)
*Nigrogranaimpatientis*	GZCC 19-0042 ^T^	MN387226	MN387228	N/A	N/A	MN389250	Zhang et al. (2020)
*Nigrogranaitalica*	MFLU 23-0139 ^T^	OR538590	OR538591	OR531365	N/A	OR531366	Bundhun et al. (2023)
*Nigrogranajinghongensis*	KUMUCC 21-0035 ^T^	MZ493303	MZ493317	MZ508421	MZ493289	MZ508412	Boonmee et al. (2021)
*Nigrogranajinghongensis*	KUMUCC 21-0036	MZ493304	MZ493318	MZ508422	MZ493290	MZ508413	Boonmee et al. (2021)
*Nigrogranakunmingensis*	ZHKUCC 22-0242 ^T^	OP456214	OP456379	N/A	OP456382	OP471608	Liu et al. (2024)
*Nigrogranakunmingensis*	ZHKUCC 22-0243	OP484334	OP456380	N/A	OP456383	OP471609	Liu et al. (2024)
*Nigrogranalincangensis*	ZHKUCC 23-0798 ^T^	OR853099	OR922323	OR966280	OR941079	OR966282	Xu et al. (2024)
*Nigrogranalincangensis*	ZHKUCC 23-0799	OR853100	OR922324	OR966281	OR941080	OR966283	Xu et al. (2024)
*Nigrogranalocuta-pollinis*	CGMCC 3.18784 ^T^	MF939601	MF939583	MF939610	N/A	MF939613	Zhao et al. (2018)
***Nigrogranalongiorostiolata***	**CGMCC 3.25626 ^T^**	**PP812421**	**PP812458**	**PP838913**	**PP812439**	**PP838945**	**In this study**
***Nigrogranalongiorostiolata***	**UESTCC 23.0200**	**PP812422**	**PP812457**	**PP838914**	**PP812440**	**PP838946**	**In this study**
*Nigrogranamackinnonii*	CBS 674.75 ^T^	KF015654	KF015612	KF015703	GQ387552	KF407986	de Gruyter et al. (2012)
*Nigrogranamagnoliae*	MFLUCC 20-0020 ^T^	MT159628	MT159622	MT159611	MT159634	MT159605	Wanasinghe et al. (2020)
*Nigrogranamagnoliae*	MFLUCC 20-0021	MT159629	MT159623	MT159612	MT159635	MT159606	Wanasinghe et al. (2020)
***Nigrogranamagnoliae***	**UESTCC 23.0203**	**PP812419**	**PP812454**	**PP838929**	**PP812437**	**PP838943**	**In this study**
***Nigrogranamagnoliae***	**CGMCC 3.25627**	**PP812420**	**PP812453**	**PP838927**	**PP812435**	**PP838931**	**In this study**
***Nigrogranamagnoliae***	**UESTCC 23.0190**	**PP812417**	**PP812456**	**PP838930**	**PP812438**	**PP838944**	**In this study**
***Nigrogranamagnoliae***	**UESTCC 23.0206**	**PP812418**	**PP812455**	**PP838928**	**PP812436**	**PP838932**	**In this study**
*Nigrogranamycophila*	CBS 141478 ^T^	KX650553	N/A	N/A	N/A	KX650526	Jaklitsch and Voglmayr (2016)
*Nigrogranamycophila*	CBS 141483	KX650555	N/A	KX650577	KX650510	KX650528	Jaklitsch and Voglmayr (2016)
***Nigrogrananeriicola***	**CGMCC 3.25624 ^T^**	**PP812430**	**PP812467**	**PP838921**	**PP812447**	**PP838937**	**In this study**
***Nigrogrananeriicola***	**UESTCC 23.0195**	**PP812429**	**PP812465**	**PP838922**	**PP812448**	**PP838938**	**In this study**
*Nigrogrananorvegica*	CBS 141485 ^T^	KX650556	N/A	KX650578	KX650511	N/A	Jaklitsch and Voglmayr (2016)
*Nigrogranaobliqua*	CBS 141477 ^T^	KX650560	N/A	KX650580	N/A	KX650531	Jaklitsch and Voglmayr (2016)
*Nigrogranaobliqua*	CBS 141475	KX650558	N/A	KX650579	KX650512	KX650530	Jaklitsch and Voglmayr (2016)
*Nigrogranaoleae*	CGMCC 3.24423 ^T^	OR253080	OR253232	N/A	N/A	OR262140	Li et al. (2023)
***Nigrogranaoleae* (*N.trachycarpi*)**	**GMB0499**	**OR120437**	**N/A**	**N/A**	**N/A**	**OR150024**	Hu et al. (2023); **In this study**
***Nigrogranaoleae (N.trachycarpi)***	**GMB0505**	**OR120440**	**N/A**	**N/A**	**N/A**	**OR150025**	Hu et al. (2023); **In this study**
***Nigrogranaoleae***	**UESTCC 23.0209**	**PP812424**	**PP812463**	**PP838915**	**PP812441**	**PP838933**	**In this study**
***Nigrogranaoleae***	**UESTCC 23.0193**	**PP812423**	**PP812459**	**PP838916**	**PP812442**	**PP838934**	**In this study**
*Nigrogranaperuviensis*	CCF 4485 ^T^	LN626658	LN626683	LN626665	LN626677	LN626671	Kolařík (2018)
*Nigrogranapuerensis*	ZHKUCC 22-0212 ^T^	OP450969	OP450975	N/A	OP450983	OP432249	Lu et al. (2022)
*Nigrogranarhizophorae*	MFLUCC 18-0397 ^T^	MN047085	N/A	MN431489	N/A	MN077064	Dayarathne et al. (2020)
*Nigrogranarubescens*	CHEM 2344 ^T^	OQ400924	OQ400934	OQ413082	N/A	OQ413077	Mack et al. (2024)
*Nigrogranasamueliana*	NFCCI 4383 ^T^	MK358817	MK358812	MK330939	MK358810	MK330937	Dayarathne et al. (2020)
*Nigrogranaschinifolii*	GMB0498 ^T^	OR120434	N/A	N/A	N/A	OR150022	Hu et al. (2023)
*Nigrogranaschinifolii*	GMB0504	OR120441	N/A	N/A	N/A	OR150023	Hu et al. (2023)
*Nigrogranasichuanensis*	CGMCC 3.24424 ^T^	OR253096	OR253248	N/A	N/A	OR251058	Li et al. (2023)
*Nigrogranathailandica*	MFLUCC 17-2663	MK762709	MK762716	N/A	MK762704	N/A	Senanayake et al. (2023)
*Nigrogranathymi*	MFLUCC 14-1096 ^T^	KY775576	KY775573	N/A	KY775574	KY775578	Hyde et al. (2017)
***Nigrogranathymi***	**UESTCC 23.0210**	**PP812428**	**PP812464**	**PP838919**	**PP812445**	**N/A**	**In this study**
***Nigrogranathymi***	**UESTCC 23.0194**	**PP812427**	**PP812462**	**PP838920**	**PP812446**	**N/A**	**In this study**
*Nigrogranaverniciae*	CGMCC 3.24425	OR253116	OR253275	N/A	N/A	OR251168	Li et al. (2023)
*Nigrogranawuhanensis*	ZHKUCC 22-0329 ^T^	OP941389	OP941390	N/A	OQ061465	OP947079	Shu et al. (2023)
*Nigrogranayasuniana*	YU 101026 ^T^	HQ108005	LN626684	LN626664	LN626676	LN626670	Kolařík (2018)
*Occultibambusabambusae*	MFLUCC 13-0855 ^T^	KU940123	KU863112	KU940170	N/A	KU940193	Dai et al. (2017)
*Occultibambusapustula*	MFLUCC 11-0502 ^T^	KU940126	KU863115	N/A	N/A	N/A	Dai et al. (2017)

* **Remarks**: The superscript T denotes ex-type isolates. “N/A” denotes sequence is unavailable. The newly generated sequences, new species and synonymized isolates are indicated in black bold font. **Abbreviations: CBS**: CBS−KNAW Fungal Biodiversity Centre, Utrecht, The Netherlands; **CCF**: Culture Collection of Fungi, Charles University, Prague, Czech Republic; **CGMCC**: China General Microbiological Culture Collection Center, Institute of Microbiology, Chinese Academy of Sciences, Beijing, China; **GMB**: Herbaria of Guizhou Medical University, Guiyang, China; **GZCC**: Guizhou Culture Collection, Guizhou, China; **KUMUCC**: Kunming Medical University Culture Collection, Kunming, China; **MFLUCC**: Mae Fah Luang University Culture Collection, Chiang Rai, Thailand; **NFCCI**: National Fungal Culture Collection of India, India; **UESTCC**: University of Electronic Science and Technology Culture Collection, Chengdu, China; **YU**: Yale University Herbarium, Connecticut, America; **ZHKUCC**: Zhongkai University of Agriculture and Engineering Culture Collection, Guangzhou, China; **Personal collections**: **ZY** and **CHEM**: These numbers are assigned by the author and have no annotations.

ML analyses were performed with RAxML-HPC v.8 on XSEDE (8.2.12) (Stamatakis 2006; Stamatakis et al. 2008) through the CIPRES Science Gateway V. 3.3 (https://www.phylo.org/portal2/login!input.action) (Miller et al. 2010). The tree search included 1,000 non-parametric bootstrap replicates; the best scoring tree was selected among suboptimal trees from each run by comparing likelihood scores under the GTRGAMMA substitution model. The resulting replicates were plotted onto the best scoring tree obtained previously. ML bootstrap values equal to or greater than 75% were marked near each node.

BI was performed in MrBayes 3.2.6 (Ronquist et al. 2012). The program MrModeltest 2 v. 2.3 (Nylander 2008) was used to determine the best nucleotide substitution model for each data partition. The evolutionary model of SYM+I+G substitution model was selected for ITS, HKY+G substitution model was selected for SSU, and GTR+I+G substitution model was selected for LSU, *rpb2* and *tef1-α*. Posterior probabilities (PP) (Rannala and Yang 1996) were determined by Markov chain Monte Carlo sampling (MCMC). Six simultaneous Markov chains were run for 10 million generations, and trees were sampled every 1,000 th generation. The first 25% of saved trees, representing the burn-in phase of the analysis, were discarded. The remaining trees were used for calculating posterior probabilities in the majority rule consensus tree (Larget and Simon 1999). PP values equal to or greater than 0.95 were marked near each node.

Phylogenetic trees were printed with Fig. Tree v. 1.4.4 (http://tree.bio.ed.ac.uk/software/figtree/) and the layout was created in Adobe Illustrator CS6 software (Adobe Systems, USA). The new sequences generated in this study were deposited in GenBank (Table 1).

### ﻿Phylogenetic results

**Figure 1.**
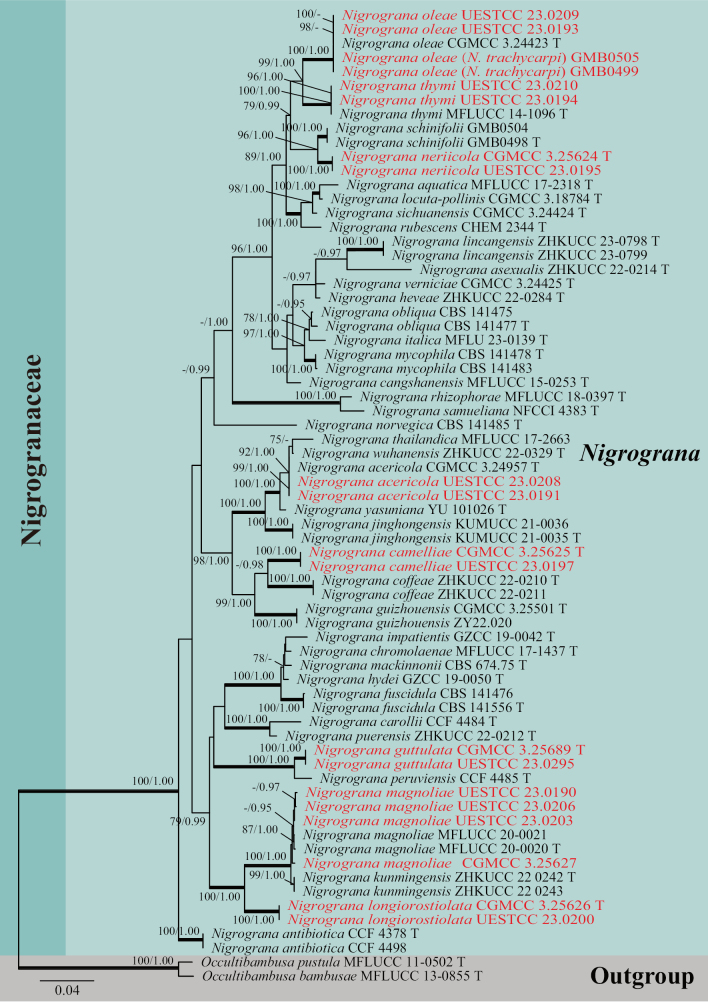
Phylogenetic tree constructed from maximum likelihood (RAxML) analyses of a combined ITS, LSU, *rpb2*, SSU, and *tef1-α* sequence data for selected genera within the family Nigrogranaceae (Pleosporales, Dothideomycetes). Branches support for Maximum likelihood (MLBS) equal to or greater than 75% and Bayesian inference posterior probabilities (BIPP) equal to or greater than 0.95 are marked above or below nodes as MLBS/BIPP. The abbreviation T indicates the ex-type strain. Species names and culture collections in red are newly collected taxa and synonymized isolates. The tree was rooted with *Occultibambusapustula* (MFLUCC 11-0502) and *O.bambusae* (MFLUCC 13-0855).

In this study, five loci, ITS, LSU, *rpb2*, SSU, and *tef1-α*, were used to determine the phylogenetic placement of the new collections. The concatenated matrix was comprised of 69 taxa with a total of 4,236 bp characters (ITS: 1–473 bp; LSU: 474–1,306 bp; *rpb2*: 1,307–2,331 bp; SSU: 2,332–3,335 bp; *tef1-α*: 3,336–4,236 bp) including gaps. Single-locus analyses were carried out to compare the topologies and clade stabilities, respectively. The results showed that ML and BI were similar in topology without significant conflicts. The best RAxML tree with a final likelihood value of -20,464.246121 is presented in Fig. 1. RAxML analysis yielded 1,200 distinct alignment patterns and 26.42% of undetermined characters or gaps. Estimated base frequencies were as follows: A = 0.247595, C = 0.247214, G = 0.264771, T = 0.240420, with substitution rates AC = 1.651782, AG = 5.572876, AT = 1.491922, CG = 1.179397, CT = 11.546850, GT = 1.000000; gamma distribution shape parameter alpha = 0.139617. Tree-Length = 1.453662. The final average standard deviation of split frequencies at the end of total MCMC generations for BI analysis was 0.009978 (the critical value for the topological convergence diagnostic is below 0.01).

Representatives of all the species of *Nigrograna* were including in our phylogenetic analysis (Fig. 1). Four strains (CGMCC 3.25627, UESTCC 23.0203, UESTCC 23.0190 and UESTCC 23.0206) were nested with *N.magnoliae* (ex-type strain MFLUCC 20-0020 and MFLUCC 20-0021), and strains, UESTCC 23.0208 and UESTCC 23.0191, UESTCC 23.0210 and UESTCC 23.0194, clustered with *N.acericola* (ex-type strain, CGMCC 3.24957) and *N.thymi* (ex-type strain, MFLUCC 14-1096), respectively. *Nigrogranatrachycarpi* (GMB0499 and GMB0505) was synonymized under *N.oleae*, these two strains of *N.trachycarpi* and our two isolates (UESTCC 23.0209 and UESTCC 23.0193) grouped with ex-type strain of *N.oleae* (CGMCC 3.24423) with maximum support (100% MLBS/1.00 BIPP).

*Nigrogranacamelliae* (CGMCC 3.25625 and UESTCC 23.0197) and *N.guttulata* (CGMCC 3.25689 and UESTCC 23.0295) were sister to *N.coffeae* (ex-type strain ZHKUCC 22-0210 and ZHKUCC 22-0211) and *N.peruviensis* (ex-type strain CCF 4485), respectively. They formed two distinct clades with 100% MLBS/1.00 BIPP and 63% MLBS/0.98 BIPP, respectively. *Nigrogrananeriicola* (CGMCC 3.25624 and UESTCC 23.0195) was sister to *N.schinifolii* (ex-type strain GMB0498 and GMB0504) and formed a strongly supported monophyletic lineage (96% MLBS/1.00 BIPP). *Nigrogranalongiorostiolata* (CGMCC 3.25626 and UESTCC 23.0200) formed a distinct lineage with high bootstrap support (100% MLBS/1.00 BIPP).

## ﻿Taxonomy

### ﻿ Nigrograna magnoliae

Taxon classificationFungiPleosporalesNigrogranaceae

Wanas, PLoS One, 15(7): 10 (2020)

F6E36AFF-128F-5278-9552-D894CF322252

557331

Fig. 2

#### Description.

***Saprobic*** on dead branches of *Buxussinica* (Buxaceae). **Sexual morph: *Ascomata*** 204–326 μm wide, 140–220 μm high (x̅ = 248 × 187 μm, n = 20), solitary or gregarious, scattered, immersed to semi-immersed, with only ostiolar necks visible on the host surface, trigonoid, uniloculate, perithecioid, globose to subglobose, brown to dark brown, with an ostiole. ***Ostiole*** central or eccentric, brittle. ***Peridium*** 15–23 μm (x̅ = 18 μm, n = 20) composed of angular cells, consisting 4–5 layers, brown to dark brown thick-walled cells of outer layer, hyaline to subhyaline thin-walled cells of inner layer. ***Hamathecium*** 1–3 μm (x̅ = 2 μm, n = 20) wide, composed of numerous, filamentous, hyaline, aseptate or separate, rarely branched, smooth-walled pseudoparaphyses. ***Asci*** 57–103 × 8–11 μm (x̅ = 72.5 × 10 μm, n = 30), 8-spored, bitunicate, fissitunicate, clavate to long cylindric-clavate, short cylindrical pedicellate with a swollen base, apically rounded, with a minute ocular chamber. ***Ascospores*** 13–19 × 4.5–6 μm (x̅ = 14.5 × 5 μm, n = 50), 1–2-seriate, partially overlapping, fusoid to ellipsoid, tapering towards the blunt ends, or blunt at both ends, guttulate, smooth-walled, olivaceous to yellowish-brown when young, 1-septate; deeply constricted at septa, becoming 3-septate, brown to dark brown when mature, without appendages. **Asexual morph**: Undetermined.

#### Culture characteristics.

Ascospores germinated on PDA within 24 h, and germ tubes produced from basal cell. Colonies growing on PDA reached 32−33 mm in diameter after three weeks at 25 °C in dark, white in the whole colony from above, and slightly raised in the center, circular, flat, edge entire, margin well-defined; in reverse, grayish black in the center, off-white at the margin, the color gradually lightens from center to edge, no pigmentation on PDA.

**Figure 2.**
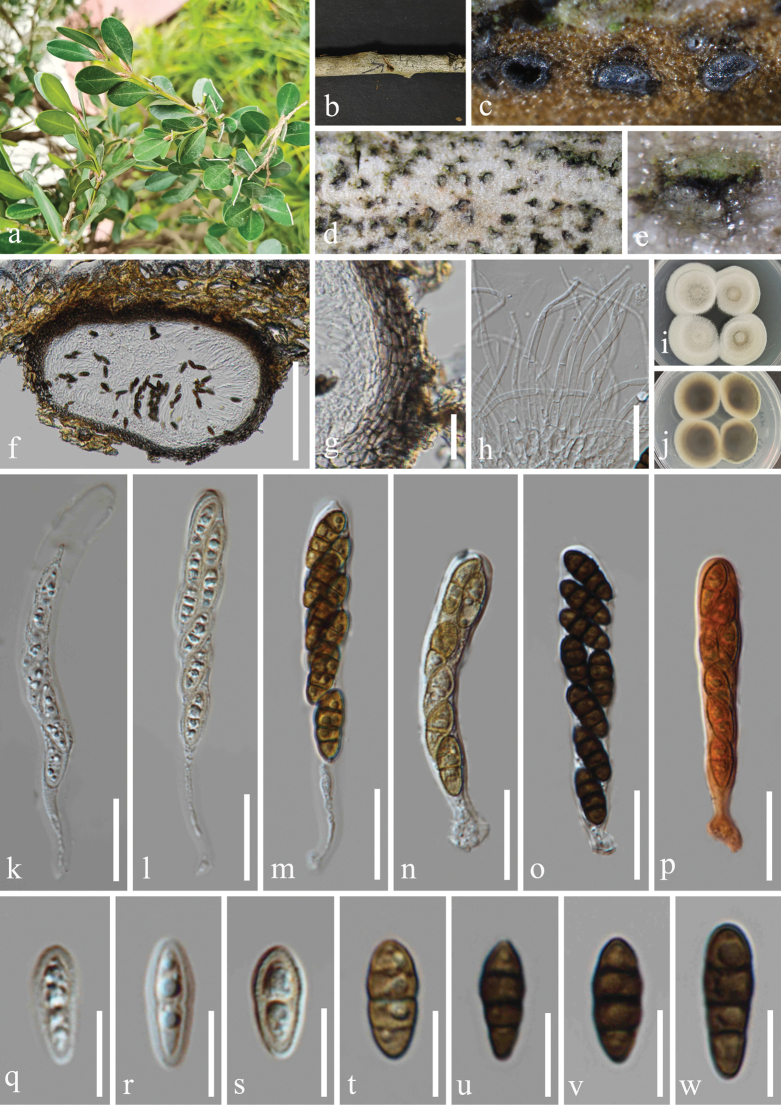
*Nigrogranamagnoliae* (HUEST 23.0203, new host record) **a** host *Buxussinica***b** branch of *Buxussinica***c–e** appearance of ascomata on host surface **f** vertical section through ascoma **g** peridium **h** hamathecium **i, j** colonies on PDA, above (**i**) and below (**j**) **k–o** asci **p** asci in Congo red **q–w** ascospores. Scale bars: 100 µm (**f**); 20 µm (**g, h**, **k–p**); 10 µm (**q–w**).

#### Material examined.

China • Yunnan Province, Kunming City, Panlong District, Kunming Botanical Garden. 25°8'27′′N, 102°44'24′′E, elevation 1,922 m, on dead branches of medicinal plant *Buxussinica* (Rehder & E. H. Wilson) M. Cheng (Buxaceae), 11 November 2022, H.Z. Du, S735 (HUEST 23.0203), living culture UESTCC 23.0203; • ibid., Sichuan Province, Chengdu City, High-tech West District, Yaobo Park, 30°43'57′′N, 103°56'21′′E, elevation 504 m, on dead branches of medicinal plant *Eucommiaulmoides* Oliv. (Eucommiaceae), 11 August 2021, H.Z. Du, S347 (HUEST 23.0206), living culture UESTCC 23.0206; • ibid., Guizhou Province, Guiyang City, Nanming District, Guiyang Medicinal Botanical Garden, 26°32'18′′N, 106°41'48′′E, elevation 1,127 m, on dead branches of medicinal plant *Mahoniabealei* (Fort.) Carr. (Berberidaceae), 12 October 2021, H.Z. Du, S370 (HUEST 23.0207), living culture CGMCC 3.25627 = UESTCC 23.0207; • ibid., Guizhou Province, Guiyang City, Huaxi District, 26°30'43"N, 106°39'32"E, elevation 1,155 m, on dead branches of medicinal plant *Camelliasinensis* (L.) O. Ktze. (Theaceae), 2 February 2023, Y.X. Yu, GY33 (HUEST 23.0190), living culture UESTCC 23.0190.

#### Notes.

*Nigrogranamagnoliae* was introduced by Wanasinghe et al. (2020) with both asexual and sexual morphs reported in China. The host distribution of this species is presented in Table 2. Our collections are identical to *N.magnoliae* based on morphology and phylogeny. Therefore, we reported it as new host records from medicinal plants of *Buxussinica*, *Camelliasinensis*, *Eucommiaulmoides* and *Mahoniabealei* in China.

**Table 2.** The host distribution of *Nigrogranamagnoliae*.

Host distribution	Collecting sites	References
*Magnoliadenudate* (Magnoliaceae)	China (Yunnan Province)	Wanasinghe et al. (2020)
Submerged wood from aquatic habitats	Thailand (Chiang Rai Province)	Zhang et al. (2020)
Decaying twigs of unidentified host	China (Guizhou Province)	Zhang et al. (2020)
*Acertruncatum* (Aceraceae)	China (Sichuan Province)	Li et al. (2023)
*Juglansregia* (Juglandaceae)	China (Sichuan Province)	Li et al. (2023)
*Oleaeuropaea* (Oleaceae)	China (Sichuan Province)	Li et al. (2023)
*Micheliaalba* (Magnoliaceae)	China (Guizhou Province)	Chethana et al. (2023)
*Rosa* sp. (Rosaceae)	China (Sichuan Province)	Chethana et al. (2023)
Fruiting bodies of *Shearia* sp. (Dothioraceae)	China (Guizhou Province)	Chethana et al. (2023)
*Magnoliagrandiflora* (Magnoliaceae)	Thailand (Chiang Mai Province)	https://www.ncbi.nlm.nih.gov/nuccore/MN081891.1
*Castanopsisindica* (Fagaceae)	China (Yunnan Province)	Ren et al. (2024)
*Buxussinica* (Buxaceae)	China (Yunnan Province)	In this study
*Eucommiaulmoides* (Eucommiaceae)	China (Sichuan Province)	In this study
*Mahoniabealei* (Berberidaceae)	China (Guizhou Province)	In this study
*Camelliasinensis* (Theaceae)	China (Guizhou Province)	In this study

### ﻿ Nigrograna longiorostiolata

Taxon classificationFungiPleosporalesNigrogranaceae

H.Z. Du & Jian K. Liu sp. nov.

4F44122A-E81C-51D2-9D09-63033DA03266

854177

Fig. 3

#### Etymology.

The epithet ‘*longiorostiolata*’ refers to the longer-ostiolate of ascomata.

#### Holotype.

HKAS 131311

#### Description.

***Saprobic*** on dead branches of *Citrusmedica* (Rutaceae). **Sexual morph: *Ascomata*** 222–293 μm wide, 144–486 μm high (x̅ = 264 × 303 μm, n = 20), solitary, scattered, immersed, visible as black dots on the host surface, uniloculate, globose to subglobose, sometimes obpyriform with a long ostiole. ***Ostioles*** 175–302 μm long, 83–128 μm wide (x̅ = 263 × 102 μm, n = 20) central or eccentric, longer, with a crest-like apex, filled with hyaline or slightly brown periphyses. ***Peridium*** 17–32 μm (x̅ = 23.5 μm, n = 20) composed of *textura prismatica* cells, consisting 3–4 layers, brown to dark brown of outer layer, hyaline to subhyaline of inner layer. ***Hamathecium*** 1–2 μm (x̅ = 1.5 μm, n = 20) wide, composed of numerous, filiform, hyaline, aseptate or separate, rarely branched, guttulate, smooth-walled pseudoparaphyses. ***Asci*** 40–70 × 6–9 μm (x̅ = 53 × 8 μm, n = 30), 5–8-spored, bitunicate, fissitunicate, clavate, short cylindrical pedicellate with a swollen base, apically rounded, with a minute ocular chamber. ***Ascospores*** 10–13 × 4–6 μm (x̅ = 12 × 5 μm, n = 50), 1–2-seriate, partially overlapping, fusoid to ellipsoid, tapering towards the blunt ends, or blunt at both ends, guttulate, olivaceous to yellowish-brown when young, aseptate or 1-septate; deeply constricted at septa, becoming 3-septate, brown to dark brown when mature, without appendages. **Asexual morph**: Undetermined.

#### Culture characteristics.

Ascospores germinated on PDA within 24 h, and germ tubes produced from basal cell. Colonies growing on PDA reached 17–18 mm in diameter after three weeks at 25 °C in dark, white in the whole colony from above, circular, edge entire, margin well-defined; in reverse, off-white to grayish brown, no pigmentation on PDA.

**Figure 3.**
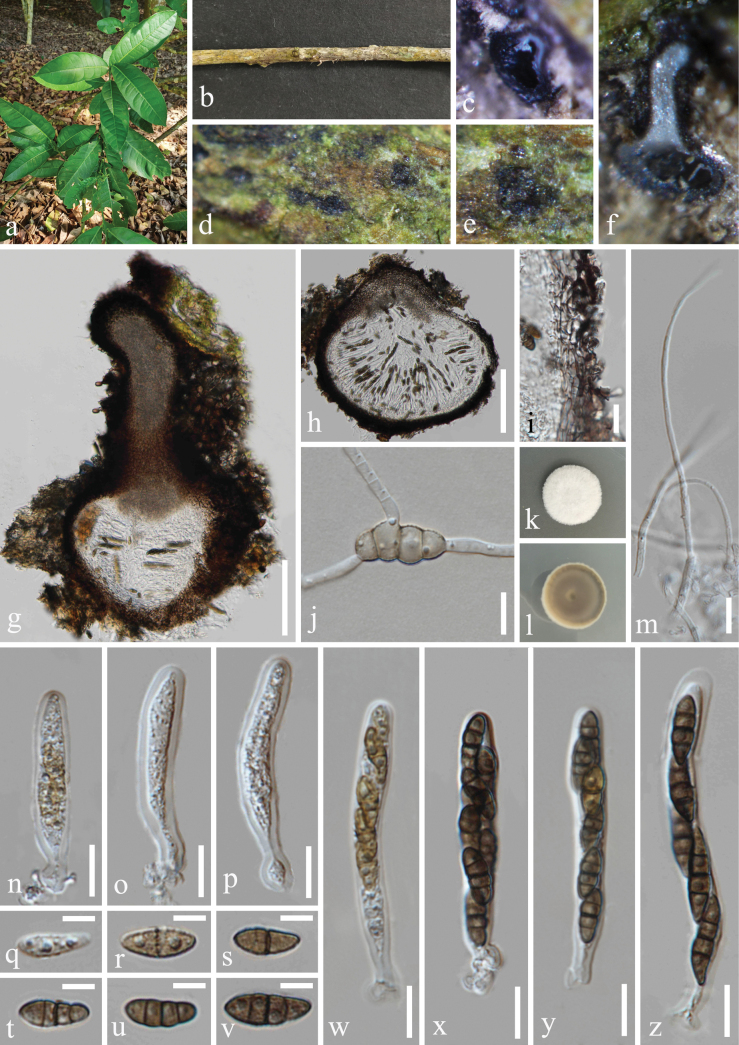
*Nigrogranalongiorostiolata* (HKAS 131311, holotype) **a** host *Citrusmedica***b** branch of *Citrusmedica***c–f** appearance of ascomata on host surface **g, h** vertical section through ascoma **i** peridium **j** germinated ascospore **k, l** colony on PDA, above (**k**) and below (**l**) **m** hamathecium **n–p, w–z** asci **q–v** ascospores. Scale bars: 100 µm (**g, h**); 10 µm (**i, j, m–p, w–z**); 5 µm (**q–v**).

#### Material examined.

China • Yunnan Province, Xishuangbanna Dai Autonomous Prefecture, Mengla County, Xishuangbanna Tropical Botanical Garden Chinese Academy of Sciences. 21°56'1′′N, 101°25'33′′E, elevation 505 m, on dead branches of medicinal plant *Citrusmedica* L. (Rutaceae), 10 November 2022, H.Z. Du, S655 (HKAS 131311, holotype; HUEST 23.0200, isotype); ex-holotype living culture CGMCC 3.25626; ex-isotype living culture UESTCC 23.0200.

#### Notes.

*Nigrogranalongiorostiolata* shares similar morphology with *N.magnoliae* (holotype, MFLU 20–0092) and *N.kunmingensis* (holotype, ZHKU 22-0141) in having immersed, globose to subglobose ascomata, bitunicate and clavate asci, fusoid to ellipsoid, 3-septate mature ascospores. However, the ascomata size of *N.longiorostiolata* (222–293 × 144–486 μm) is larger than *N.magnoliae* (200–300 × 100–150 μm) (Wanasinghe et al. 2020) and smaller than *N.kunmingensis* (300–500 × 390–450 µm) (Liu et al. 2024). The phylogenetic result (Fig. 1) showed that *N.longiorostiolata* (CGMCC 3.25626 and UESTCC 23.0200) can be recognized as a distinct phylogenetic species with high bootstrap support (100% MLBS/1.00 BIPP). Additionally, *N.longiorostiolata* (ex-type strain, CGMCC 3.25626) can be distinguished from *N.magnoliae* (ex-type strain, MFLUCC 20-0020) by 26/471 bp (5.5%, 2 gaps) in ITS, 14/831 bp (1.7%, without gaps) in LSU, 30/855 bp (3.5%, 3 gaps) in *tef1-α* and 96/1042 bp (9.2%, without gaps) in *rpb2* differences, and differs from *N.kunmingensis* (ex-type strain, ZHKUCC 22-0242) with 70/823 bp (8.5%, 21 gaps) of ITS, 14/844 bp (1.7%, without gaps) of LSU and 30/855 bp (3.5%, 3 gaps) of *tef1-α* differences. Therefore, *N.longiorostiolata* associated with *Citrusmedica* is a phylogenetically distinct specie and introduced as a new species.

### ﻿ Nigrograna acericola

Taxon classificationFungiPleosporalesNigrogranaceae

W.L. Li & Jian K. Liu, Mycosphere, 14(1): 1496–1500 (2023)

906957B3-261F-564E-A6AC-C409562E44BD

849155

Fig. 4

#### Description.

***Saprobic*** on dead branches of *Gymnosporiaacuminata* (Celastraceae). **Sexual morph: *Ascomata*** 524–647 × 341–475 μm (x̅ = 586 × 424 μm, n = 20), solitary, scattered, immersed, ostiolar necks visible on the host surface or erumpent, subglobose to ellipsoid, coriaceous, brown to dark brown, with an ostiole. ***Ostioles*** 86–138 μm long, 64–119 μm wide (x̅ = 113 × 96 μm, n = 20), mostly central, some eccentric, with a crest-like apex, central, filled with hyaline periphyses. ***Peridium*** 15–58 μm (x̅ = 40 μm, n = 20) μm wide, composed of 4–5 layers of flattened, brown to dark brown, thin-walled cells of ***textura angularis***, the inner layer is dense, the outer layer sparse. ***Hamathecium*** 1.5–3 μm (x̅ = 2 μm, n = 20) wide, composed of numerous, filamentous, hyaline, unbranched pseudoparaphyses. ***Asci*** 70–87 × 12–14 μm (x̅ = 77 × 13 μm, n = 30), 8-spored, bitunicate, fissitunicate, cylindrical to clavate, short pedicellate, apically rounded, with a minute ocular chamber. ***Ascospores*** 16–19 × 5–7 μm (x̅ = 17 × 6 μm, n = 50), 1–2-seriate, biseriate or partially overlapping, fusoid to ellipsoid, with obtuse ends, tapering towards the ends, guttulate, smooth-walled, 1-septate, subhyaline to yellowish-brown when young; becoming 3-septate, slightly constricted at the middle septum, brown to dark brown when mature, without appendages. **Asexual morph**: Undetermined.

**Figure 4.**
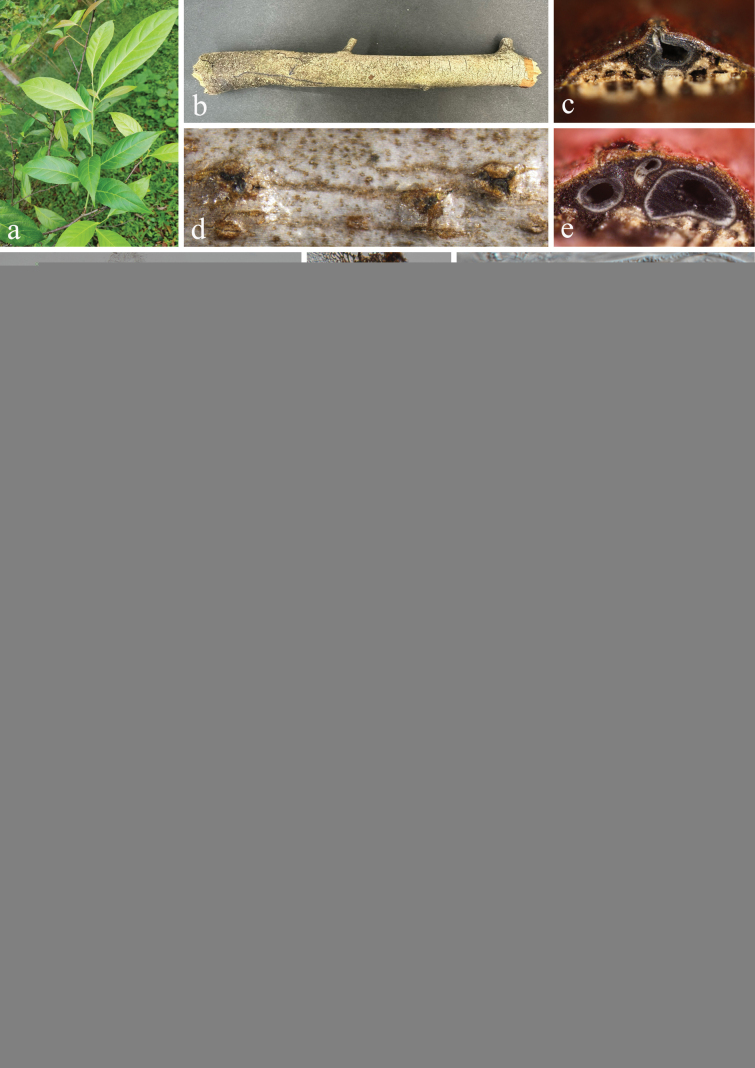
*Nigrogranaacericola* (HUEST 23.0208, new host record) **a** host *Gymnosporiaacuminata***b** branch of *Gymnosporiaacuminata***c–e** appearance of ascomata on host surface **f** vertical section through ascoma **g** peridium **h** hamathecium **i, j** colony on PDA, above (**i**) and below (**j**) **k–o** asci. **p–u** ascospores. Scale bars: 200 µm (**f**); 50 µm (**g**); 10 µm (**h, p–u**); 20 µm (**k–o**).

#### Culture characteristics.

Ascospores germinated on PDA within 24 h, and germ tubes produced from basal cell. Colonies growing on PDA reached 38–40 mm in diameter after three weeks at 25 °C in dark, white in the whole colony from above, circular, edge entire, margin well-defined; in reverse, light brown in the center, olive gray at the margin, no pigmentation on PDA.

#### Material examined.

China • Yunnan Province, Xishuangbanna Dai Autonomous Prefecture, Mengla County, Xishuangbanna Tropical Botanical Garden Chinese Academy of Sciences. 21°55'54′′N, 101°15'16′′E, elevation 511 m, on dead branches of medicinal plant *Gymnosporiaacuminata* Hook. f. (Celastraceae), 10 November 2022, H.Z. Du, D03 (HUEST 23.0208), living culture UESTCC 23.0208; *ibid*., Sichuan Province, Zigong City, Rong County, 29°29'1"N, 104°14'19"E, elevation 850 m, on dead branches of medicinal plant *Camelliasinensis* (L.) O. Ktze. (Theaceae), 3 November 2022, Y. H. Lu & Y. Xiao, CS11(HUEST 23.0191), living culture UESTCC 23.0191.

#### Notes.

*Nigrogranaacericola* was introduced by Li et al. (2023) from *Acertruncatum* (Aceraceae) in China. Our collections are identical to *N.acericola* based on morphology and phylogeny. We reported it as new host records from *Camelliasinensis* and *Gymnosporiaacuminata* in China.

### ﻿ Nigrograna camelliae

Taxon classificationFungiPleosporalesNigrogranaceae

Y.H. Lu, H.Z. Du & Jian K. Liu sp. nov.

65398308-6B0B-5489-8561-CC6B1F6C1EE3

854178

Fig. 5

#### Etymology.

The epithet ‘*camelliae*’ refers to the host genus *Camelliae* from which the fungus was originally isolated.

#### Holotype.

HKAS 131310

#### Description.

***Saprobic*** on dead branches of *Camelliasinensis* (Theaceae). **Sexual morph: *Ascomata*** 137–270 μm wide, 208–324 μm high (x̅ = 212 × 265 μm, n = 20), solitary, scattered, immersed, black spots on the host substrate, globose to subglobose, sometimes obpyriform, ostiolate, hairs of ascomata 2–3 μm wide, slightly brown, septate. ***Ostioles*** 65–138 μm long, 32–60 μm wide (x̅ = 100 × 45 μm, n = 20) mostly central, some eccentric, with a crest-like apex. ***Peridium*** 19–30 μm (x̅ = 23 μm, n = 20) wide, composed of 2–3 layers, comprising reddish brown to dark brown pigmented cells. ***Hamathecium*** 2–3 μm (x̅ = 2.5 μm, n = 20) wide, composed of numerous, filiform, hyaline, aseptate or separate, filamentous, smooth-walled pseudoparaphyses. ***Asci*** 70–108 × 9–11 μm (x̅ = 80 × 10 μm, n = 30), 8-spored, bitunicate, fissitunicate, clavate to cylindric-clavate, short stalked, some with a swollen base, apically rounded, with a small ocular chamber. ***Ascospores*** 13–16 × 4–6 μm (x̅ = 15 × 5 μm, n = 50), overlapping uni- to bi-seriately arranged, fusoid to ellipsoid, tapering towards the blunt ends, or blunt at both ends, straight or slightly curved, 1-septate, constricted, with obviously guttulate, hyaline to slightly brown when immature, pale brown to brown when mature, without appendages. **Asexual morph**: Undetermined.

**Figure 5.**
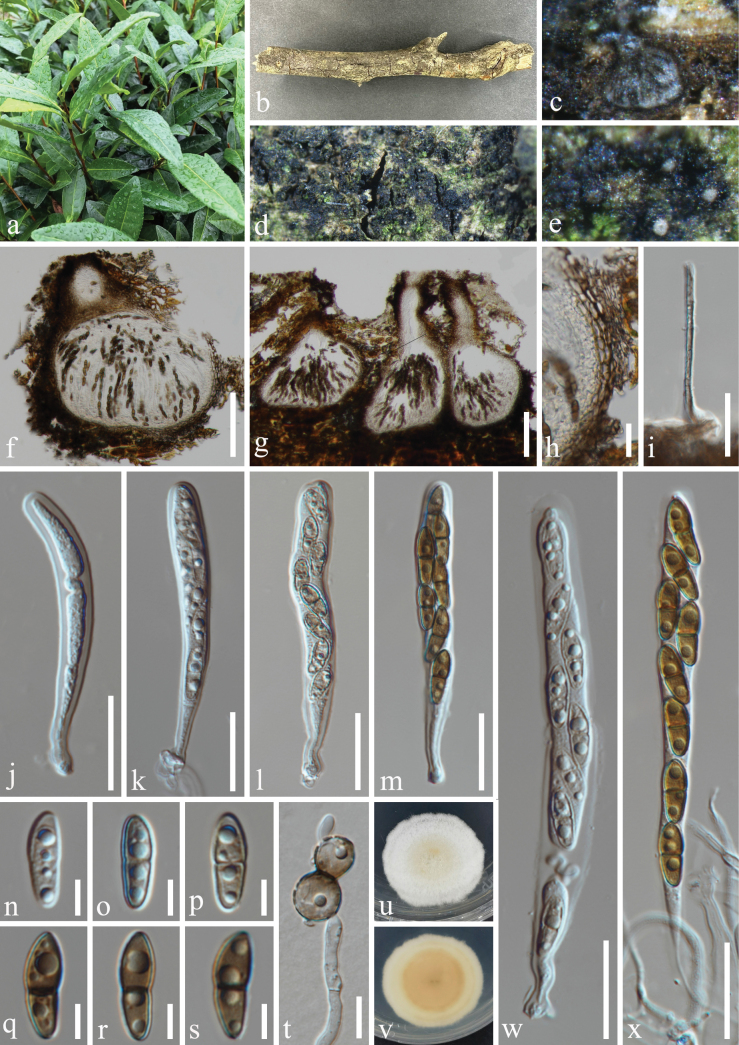
*Nigrogranacamelliae* (HKAS 131310, holotype) **a** host *Camelliasinensis***b** branch of *Camelliasinensis***c–e** appearance of ascomata on host surface **f, g** vertical section through ascoma **h** peridium **i** hairs on ascomata **j–m, w, x** asci **n–s** ascospores **t** germinated ascospore **u, v** colony on PDA, above (**u**) and below (**v**). Scale bars: 100 µm (**f, g**); 20 µm (**h–m, w, x**); 5 µm (**n–s**); 10 µm (**t**).

#### Culture characteristics.

Ascospores germinated on PDA within 24 h, and germ tubes produced from basal cell. Colonies growing on PDA reached 35–36 mm in diameter after three weeks at 25 °C in dark, white in the whole colony and slightly bright yellow in the center from above, circular, edge entire, margin well-defined; in reverse, yellowish brown in the center, slightly brown at the margin and presented an outer ring, no pigmentation on PDA.

#### Material examined.

China • Sichuan Province, Yaan City, Mingshan County, Mengding Mountain. 30°4'32"N, 103°2'23"E, elevation 1,200 m, on dead branches of medicinal plant *Camelliasinensis* (L.) O. Ktze. (Theaceae), 16 July 2023, Y. H. Lu & X. D. Liang, MD03A (HKAS 131310, holotype; HUEST 23.0197, isotype); ex-holotype living culture CGMCC 3.25625; ex-isotype living culture UESTCC 23.0197.

#### Notes.

*Nigrogranacamelliae* is phylogenetically close to *N.coffeae* and represents as a distinct lineage (Fig. 1). Additionally, the nucleotide base pair comparison between *N.camelliae* (ex-type strain, CGMCC 3.25625) and *N.coffeae* (ex-type strain, ZHKUCC 22-0210) revealed 15/514 bp (2.9%, 1 gap) of ITS, 11/698 bp (1.6%, without gaps) of LSU, 74/739 bp (10.0% without gaps) of *rpb2* and 28/914 bp (3.1%, without gaps) of *tef1-α* differences. Furthermore, *N.camelliae* morphologically resembles *N.coffeae* in having immersed ascomata, clavate and short pedicellate asci, pale brown to brown and septate ascospores with obviously guttulate (Lu et al. 2022). However, *N.camelliae* differs from *N.coffeae* in having ascomata with hairs and ostioles, solitary or scattered in the substrate. Additionally, they can be distinguished in having larger ascomata (208–324 × 137–270 µm vs. 140–200 × 90–140 µm) and asci (70–108 × 9–11 µm vs. 50–70 × 7–11 µm). Therefore, *N.camelliae* is introduced as a new species with the justification of phylogenetic and morphological evidence.

### ﻿ Nigrograna oleae

Taxon classificationFungiPleosporalesNigrogranaceae

W.L. Li & Jian K. Liu, Mycosphere, 14(1): 1503–1505 (2023)

F9EA06A1-D1C1-5A7F-BB17-A1D212B770BB

849157

Fig. 6

= Nigrogranatrachycarpi, MycoKeys 100: 141 (2023).

#### Description.

***Saprobic*** on dead branches of *Ardisiacrenata* (Primulaceae). **Sexual morph: *Ascomata*** 190–334 μm wide, 303–406 μm high (x̅ = 233 × 370 μm, n = 20), solitary or gregarious, scattered, immersed, often lying parallelly or obliquely to the bark or host surface, with a cylindrical ostiolar neck, coriaceous, obpyriform, brown to dark brown. ***Ostioles*** central or eccentric, filled with hyaline periphyses. ***Peridium*** 16.5–25 μm (x̅ = 21 μm, n = 20) wide, consisting 4–6 layers of brown-walled cells of ***textura angularis***. ***Hamathecium*** 1–2 μm (x̅ = 1.5 μm, n = 20) wide, aseptate or separate, composed of numerous, filiform, smooth-walled pseudoparaphyses. ***Asci*** 62–127 × 9–12 μm (x̅ = 82 × 10 μm, n = 30), 8-spored, bitunicate, fissitunicate, clavate to long cylindric-clavate, with a short pedicel, apically rounded, with a smaller ocular chamber. ***Ascospores*** 14–17 × 4–6 μm (x̅ = 15 × 5 μm, n = 50), 1–2-seriate, fusoid to ellipsoid, apical cell mostly obtuse, straight or slightly curved, guttulate, smooth-walled, 3-septate, constricted at the septa, pale brown to yellow-brown when young, brown to chocolate-brown at maturation, without appendages. **Asexual morph**: Undetermined.

#### Culture characteristics.

Ascospores germinated on PDA within 24 h, and germ tubes produced from basal cell. Colonies growing on PDA reached 22–23 mm in diameter after three weeks at 25 °C in dark. Colonies from above, circular, margin entire, dense, surface smooth, velvety appearance, white in the center, presented a pale greenish furrowed ring, white to cream at the margin; in reverse, brown in the central point, brown-gray in the middle, white to pale brownish at the edge, no pigmentation on PDA.

**Figure 6.**
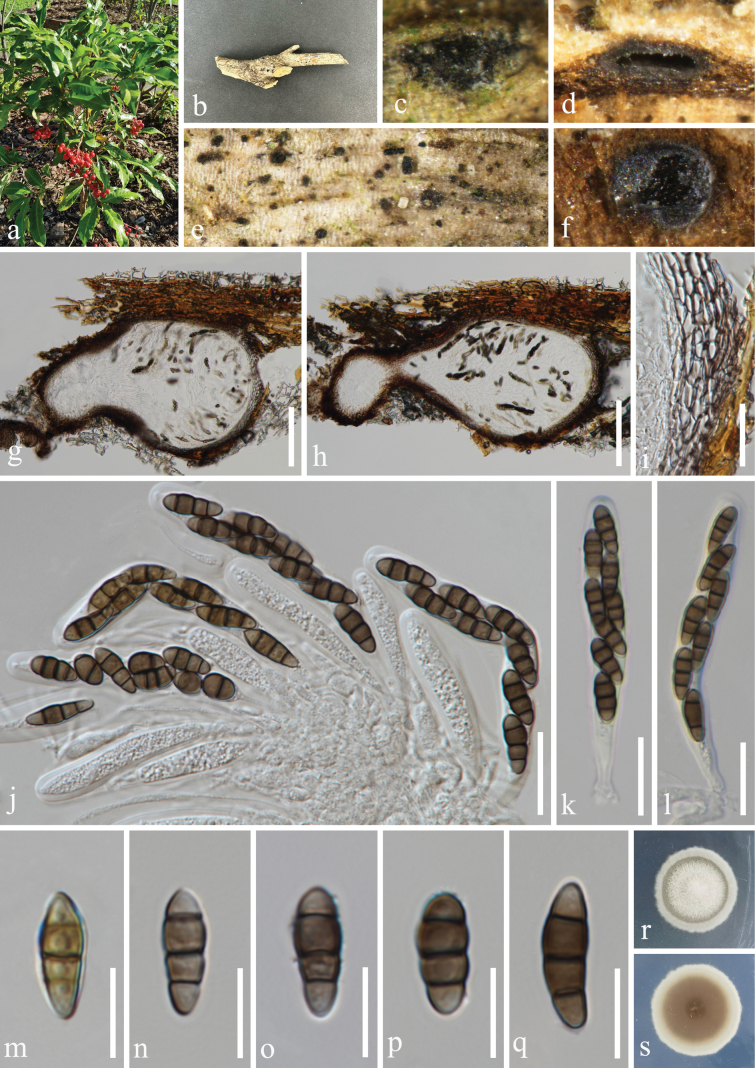
*Nigrogranaoleae* (HUEST 23.0209, new host record) **a** host *Ardisiacrenata***b** branch of *Ardisiacrenata***c–f** appearance of ascomata on host surface **g, h** vertical section through ascoma **i** peridium **j–l** asci **m–q** ascospores **r, s** colony on PDA, above (**r**) and below (**s**). Scale bars: 100 µm (**g, h**); 20 µm (**i−l**); 10 µm (**m–q**).

#### Material examined.

China • Yunnan Province, Xishuangbanna Dai Autonomous Prefecture, Mengla County, Xishuangbanna Tropical Botanical Garden Chinese Academy of Sciences. 21°55'49′′N, 101°15'19′′E, elevation 516 m, on dead branches of medicinal plant *Ardisiacrenata* Sims (Primulaceae), 10 November 2022, H.Z. Du, D01 (HUEST 23.0209), living culture UESTCC 23.0209; • ibid., Sichuan Province, Chengdu City, Pujiang County, 30°11'42"N, 103°22'21"E, elevation 630 m, on dead branches of *Camelliasinensis* (L.) O. Ktze. (Theaceae), 5 October 2022, Y.H. Lu & Y. Xiao, A11 (HUEST 23.0193), living culture UESTCC 23.0193.

#### Notes.

*Nigrogranaoleae* was introduced by Li et al. (2023) from *Oleaeuropaea* and *N.trachycarpi* was described by Hu et al. (2023) from *Trachycarpus* sp. in China. In this study, multi-locus phylogeny indicated that our two isolates clustered together with *N.oleae* (ex-type strain, CGMCC 3.24423) and *N.trachycarpi* (ex-type strain, GMB0499) by strong support (100% MLBS/1.00 BIPP) (Fig. 1). In addition, the nucleotide base pair comparison of ex-type strain between *N.oleae* (CGMCC 3.24423) and *N.trachycarpi* (GMB0499) was identical by 421/421 bp (100%) of ITS, and 466/466 bp (100%) of *tef1-α*. Additionally, our newly collected specimens share similar morphology with *N.oleae* and *N.trachycarpi*. Therefore, we identify our collections as *N.oleae* and propose the synonymy of *N.trachycarpi* under *N.oleae* based on morphology and phylogeny. The new host records for *N.oleae* from medicinal plants *Ardisiacrenata* and *Camelliasinensis* are reported in this study.

### ﻿ Nigrograna thymi

Taxon classificationFungiPleosporalesNigrogranaceae

Mapook, Camporesi & K.D. Hyde, Fungal Diversity, 87: 68–70 (2017)

88F3D4B1-F1E4-5BAB-818C-06E2C5DA9BA1

552958

Fig. 7

#### Description.

***Saprobic*** on dead branches of *Huangtciarenifolia* (Fabaceae). **Sexual morph: *Ascomata*** 292–359 μm wide, 166–278 μm high (x̅ = 327 × 218 μm, n = 20), solitary or scattered, immersed or semi-immersed to slightly erumpent through host tissue, coriaceous, globose to subglobose, brown to dark brown, hairs of ascomata 2–3 μm wide, brown, septate, branched. ***Ostiole*** inconspicuous, without papillate. ***Peridium*** 15–44 μm (x̅ = 29.5 μm, n = 20) wide, 5–6 layers, comprising dark brown cells of ***textura angularis***. ***Hamathecium*** comprising 1–3 μm (x̅ = 2 μm, n = 20) wide, cylindrical to filiform, septate, branched, smooth-walled pseudoparaphyses. ***Asci*** 43–86 × 7–9 μm (x̅ = 66 × 8 μm, n = 30), 8-spored, bitunicate, cylindrical to broadly filiform, with small ocular chamber. ***Ascospores*** 11–15 × 4–6 μm (x̅ = 13 × 4.5 μm, n = 50), 1–2-seriate, overlapping, broadly fusiform to inequilateral, widest at the middle cell, guttulate, smooth-walled, aseptate or 1-septate, hyaline when immature, becoming 3-septate, slightly constricted at the septum, pale brown to brown at maturity, without appendages. **Asexual morph**: Undetermined.

#### Culture characteristics.

Ascospores germinated on PDA within 24 h, and germ tubes produced from basal cell. Colonies growing on PDA reached 20 mm in diameter after three weeks at 25 °C in dark. Colonies from above, white in the whole colony and raised in the center, circular, edge entire, margin well-defined; in reverse, grayish-green in the center, white to pale green ring at the margin, no pigmentation on PDA.

**Figure 7.**
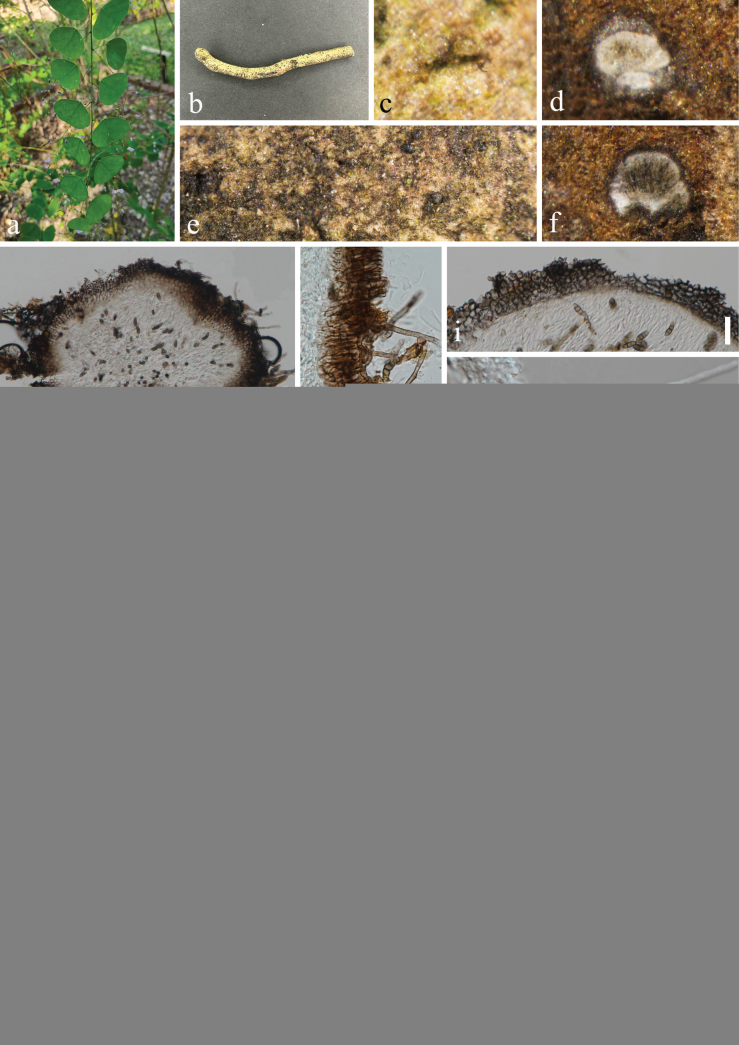
*Nigrogranathymi* (HUEST 23.0210, new host record) **a** host *Huangtciarenifolia***b** branch of *Huangtciarenifolia***c–f** appearance of ascomata on host surface **g** vertical section through ascoma **h** hairs on ascomata **i** peridium **j** hamathecium **k–o** asci **p** germinated ascospore **q, r** colony on PDA, above (**q**) and below (**r**) **s–x** ascospores. Scale bars: 100 µm (**g**); 20 µm (**h, i, l–p**); 10 µm (**j, k, s–x**).

#### Material examined.

China • Yunnan Province, Xishuangbanna Dai Autonomous Prefecture, Mengla County, Xishuangbanna Tropical Botanical Garden Chinese Academy of Sciences. 21°55'50′′N, 101°15'29′′E, elevation 515 m, on dead branches of medicinal plant *Huangtciarenifolia* (L.) H. Ohashi & K. Ohashi (Fabaceae), 10 November 2022, H.Z. Du, D02 (HUEST 23.0210), living culture UESTCC 23.0210; • ibid., Sichuan Province, Leshan City, Emeishan County, 29°36'10"N, 103°21'54"E, elevation 1,100 m, on dead branches of *Camelliasinensis* (L.) O. Ktze. (Theaceae), 18 July 2023, Y.H. Lu & X.D. Liang, EM03 (HUEST 23.0194), living culture UESTCC 23.0194.

#### Notes.

*Nigrogranathymi* was introduced by Hyde et al. (2017) from *Thymusoenipontanus* in Italy. Our collections are identical to *N.thymi* based on morphology and phylogeny (Fig. 1). We reported it as new host records from medicinal plants *Huangtciarenifolia* and *Camelliasinensis* in China.

### ﻿ Nigrograna neriicola

Taxon classificationFungiPleosporalesNigrogranaceae

Y.H. Lu, H.Z. Du & Jian K. Liu sp. nov.

E3BFFA32-7733-5F74-B8E9-883B3EEFC7AB

854179

Fig. 8

#### Etymology.

The epithet ‘*neriicola*’ refers to the host genus *Nerium* from which the fungus was originally isolated.

#### Holotype.

HKAS 131313.

#### Description.

***Saprobic*** on dead branches of *Neriumoleander* (Apocynaceae). **Sexual morph: *Ascomata*** 138–231 μm wide, 156–251 μm high (x̅ = 182 × 202 μm, n = 20), mostly gregarious, sometimes solitary, scattered, immersed to semi-immersed, appearing as black irregular protrusions and cracks, globose to subglobose, sometimes obpyriform, dark brown to black, with an ostiole. ***Ostioles*** 32–54 μm long, 14–34 μm wide (x̅ = 45 × 25 μm, n = 20) mostly central, some eccentric, with a crest-like apex. ***Peridium*** 16–61 μm (x̅ = 32 μm, n = 20) wide, multi-layered, reticulate structure, comprising dark brown to reddish brown pigmented cells of ***textura angularis***. ***Hamathecium*** 1–2.5 μm wide (x̅ = 2 μm, n = 20), composed of numerous, filiform, hyaline, aseptate or separate, rarely branched, filamentous, smooth-walled pseudoparaphyses. ***Asci*** 35–80 × 7–10 μm (x̅ = 56 × 8.5 μm, n = 30), 8-spored, bitunicate, fissitunicate, clavate to cylindric-clavate, short stalked, some with club-shape pedicel, apically rounded with a small ocular chamber. ***Ascospores*** 12–21(–31) × 3.5–5 μm (x̅ = 16 × 4 μm, n = 50), uni- to bi-seriately arranged, partially overlapping, fusoid to ellipsoid, tapering towards the blunt ends, or blunt at both ends, straight or slightly curved, guttulate, smooth-walled, 1-septate, subhyaline to slightly brown when young; becoming 3-septate, yellowish-brown to dark brown when mature, deeply constricted at septa, without appendages. **Asexual morph**: Undetermined.

#### Culture characteristics.

Ascospores germinated on PDA within 24 h, and germ tubes produced from basal cell. Colonies growing on PDA reached 33–35 mm in diameter after one month at 25 °C in dark, slightly brown in the whole colony and raised in the central point from above, circular, edge entire, margin well-defined, aerial mycelia dense; in reverse, black-brown in the center, slightly brown ring at the margin, no pigmentation on PDA.

**Figure 8.**
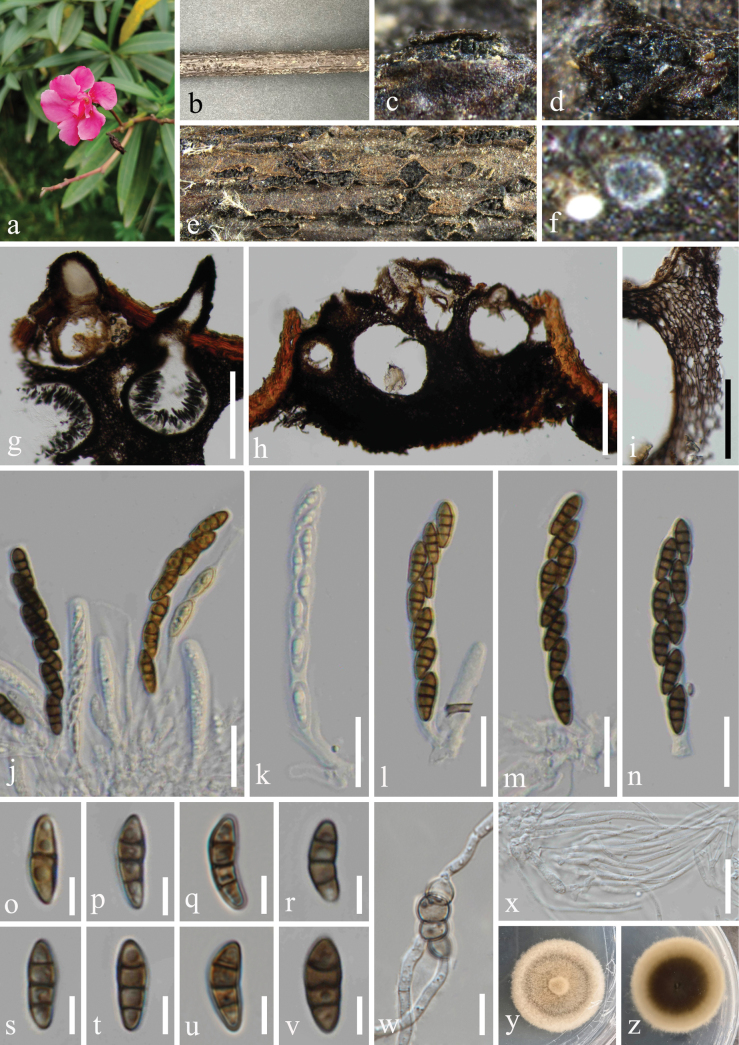
*Nigrogrananeriicola* (HKAS 131313, holotype) **a** host *Neriumoleander***b** branch of *Neriumoleander***c–f** appearance of ascomata on host surface **g, h** vertical section through ascoma **i** peridium **j–n** asci **o–v** ascospores **w** germinated ascospore **x** hamathecium **y, z** colony on PDA, above (**y**) and below (**z**). Scale bars: 200 µm (**g, h**); 100 µm (**i**); 20 µm (**j–n, x**); 5 µm (**o–v**); 10 µm (**w**).

#### Material examined.

China • Yunnan Province, Xishuangbanna Dai Autonomous Prefecture, Mengla County, Xishuangbanna Tropical Botanical Garden Chinese Academy of Sciences. 21°55'52′′N, 101°15'29′′E, elevation 505 m, on dead branches of medicinal plant *Neriumoleander* L. (Apocynaceae), 10 November 2022, H.Z. Du, D04 (HKAS 131313, holotype); ex-holotype living culture CGMCC 3.25624; • ibid., Sichuan Province, Chengdu City, Pujiang County. 30°11'40"N, 103°22'25"E, elevation 600 m, on dead branches of *Camelliasinensis* (L.) O. Ktze. (Theaceae), 5 October 2022, Y.H. Lu & Y. Xiao, M03 (HUEST 23.0195, paratype); ex-paratype living culture UESTCC 23.0195.

#### Notes.

*Nigrogrananeriicola* (CGMCC 3.25624 and UESTCC 23.0195) has close phylogenetic relationships with *N.schinifolii* (GMB0498 and GMB0504) but formed a distinct lineage (Fig. 1). Morphologically, the ascomata of *N.neriicola* differs from *N.schinifolii* in having black irregular protrusions and cracks, mostly gregarious, and ascospores that are slightly larger than *N.schinifolii* (12–21 × 3.5–5 μm vs. 10–14 × 2.8–4 μm) (Hu et al. 2023). Additionally, the nucleotide base pair comparison between *N.neriicola* (ex-type strain, CGMCC 3.25624) and *N.schinifolii* (ex-type strain, GMB0498) revealed no significant differences by 375/377 bp (99.5%, 1 gap) of ITS and 507/511 bp (99.2%, without gaps) of *tef1-α*. However, for *tef1-α* gene, the length of the two *N.schinifolii* strains (GMB0498 and GMB0504) is only 511 bp. The problem of low similarity occurred after the blastn search without a corresponding sequence in the same genus for alignment. Therefore, *N.neriicola* is introduced as a new species with the morpho-molecular data analysis.

### ﻿ Nigrograna guttulata

Taxon classificationFungiPleosporalesNigrogranaceae

Y.H. Lu, H.Z. Du & Jian K. Liu sp. nov.

2CC97985-4550-5514-85E2-C1D0EF362C63

854180

Fig. 9

#### Etymology.

The epithet ‘*guttulata*’ refers to the guttulate ascospores.

#### Holotype.

HKAS 131992.

#### Description.

***Saprobic*** on dead branches of *Camelliasinensis* (Theaceae). **Sexual morph: *Ascomata*** 182–283 μm wide, 106–276 μm high (x̅ = 241 × 183 μm, n = 20), solitary, immersed, ostiolar necks visible on the host surface or erumpent, triangular, globose to subglobose, sometimes obpyriform, coriaceous, ostiolate, dark brown to black. ***Ostioles*** 35–61 μm long, 15–30 μm wide (x̅ = 47 × 22 μm, n = 20) mostly central, some eccentric, filled with hyaline periphyses. ***Peridium*** 15–37 μm (x̅ = 25 μm, n = 20) wide, multi-layered, reticulate structure, comprising dark brown to reddish brown pigmented cells of ***textura angularis***. ***Hamathecium*** 1–2.5 μm wide (x̅ = 2 μm, n = 20), composed of numerous, filiform, hyaline, aseptate or separate, some branched, filamentous, smooth-walled pseudoparaphyses. ***Asci*** 35–70 × 7–12 μm (x̅ = 48 × 8.5 μm, n = 30), 8-spored, bitunicate, fissitunicate, clavate to cylindric-clavate, short stalked, some with club-shape pedicel, apically rounded, with small ocular chamber. ***Ascospores*** 10–13 × 3–5 μm (x̅ = 12 × 4 μm, n = 50), 1–2-seriate, overlapping, fusoid to ellipsoid, tapering towards the blunt ends, or blunt at both ends, straight or slightly curved, guttulate, smooth-walled, subhyaline to slightly brown when young, 1-septate; yellowish-brown to dark brown when mature, becoming 3-septate, deeply constricted at septa, without appendages. **Asexual morph**: Undetermined.

**Figure 9.**
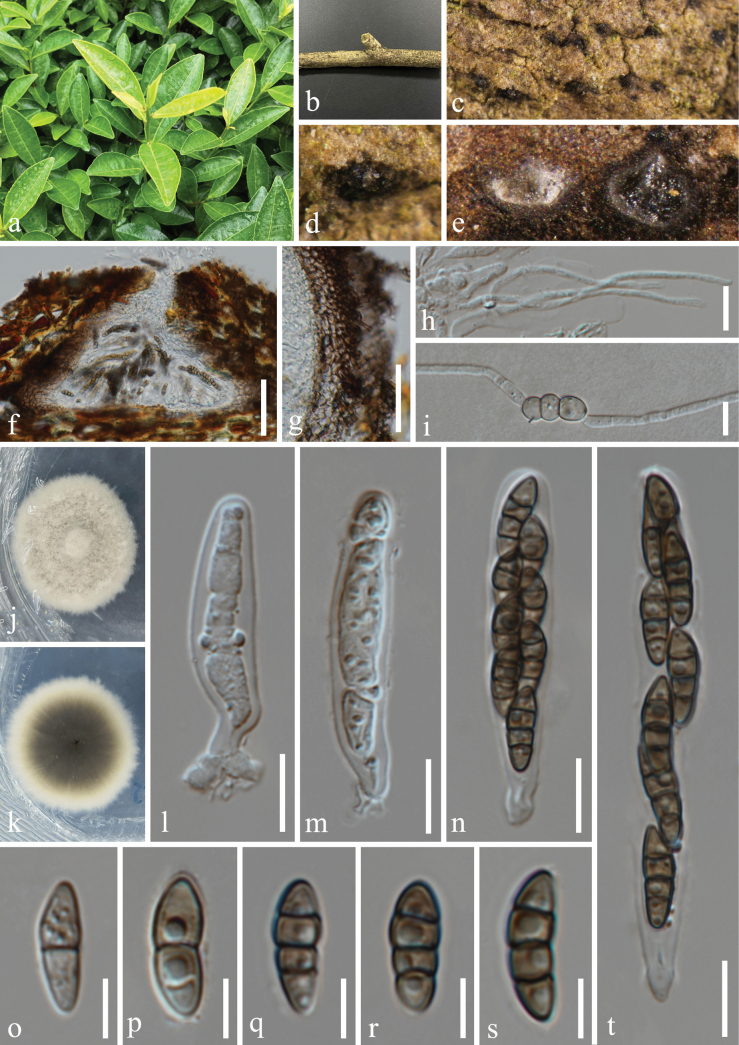
*Nigrogranaguttulata* (HKAS 131992, holotype) **a** host *Camelliasinensis***b** branch of *Camelliasinensis***c–e** appearance of ascomata on host surface **f** vertical section through ascoma **g** peridium **h** hamathecium **i** germinated ascospore **j, k** colony on PDA, above (**j**) and below (**k**) **l–n, t** asci **o–s** ascospores. Scale bars: 50 µm (**f**); 40 µm (**g**); 10 µm (**h, i, l–n, t**); 5 µm (**o–s**).

#### Culture characteristics.

Ascospores germinated on PDA within 24 h, and germ tubes produced from basal cell. Colonies growing on PDA reached 35–38 mm in diameter after one month at 25 °C in dark. Colonies from above, white in the whole colony and raised in the central point, circular, margin well-defined, aerial mycelia dense; in reverse, grayish green in the center, white ring at the margin, no pigmentation on PDA.

#### Material examined.

China • Guizhou Province, Guiyang City, Huaxi District, 26°30'40"N, 106°39'30"E, elevation 1,155 m, on dead branches of medicinal plant *Camelliasinensis* (Linnaeus) Kuntze (Theaceae), 2 February 2023, Y.X. Yu & Y.H. Lu, GY15 (HKAS 131992, holotype; HUEST 23.0295, isotype), ex-holotype living culture CGMCC 3.25689; ex-isotype living culture UESTCC 23.0295.

#### Notes.

*Nigrogranaperuviensis* was reported by Kolařík et al. (2017) as an endophytic fungus (*Biatriosporaperuviensis*) and was synonymized under the genus *Nigrograna* by Kolařík (2018), but with a lack of detailed morphological structures. In this study, our isolates of *N.guttulata* (CGMCC 3.25689 and UESTCC 23.0295) have a close phylogenetic relationship with *N.peruviensis* (Kolařík et al. 2017; Kolařík 2018) based on ITS, LSU, *rpb2*, SSU, and *tef1-α* sequence data, and formed a distinct lineage with absolute bootstrap support (100% MLBS/1.00 BIPP) (Fig. 1). Additionally, *N.guttulata* (ex-type strain, CGMCC 3.25689) can be distinguished from *N.peruviensis* (ex-type strain, CCF 4485) by 8/462 bp (1.7%, 3 gaps) in ITS, 24/1020 bp (2.4%, without gaps) in LSU and 10/618 bp (1.6%, without gaps) in *rpb2* differences. Therefore, the establishment of the new species *N.guttulata* is justified by the phylogenetic evidence.

## ﻿Discussion

In this study, eighteen isolates of *Nigrograna* (Nigrogranaceae, Pleosporales, Dothideomycetes) were obtained from medicinal plants in Southwest China (Guizhou, Sichuan and Yunnan Provinces). Based on morphological and culture characteristics, and phylogenetic analyses of combined ITS, LSU, *rpb2*, SSU, and *tef1-α* sequence data, four novel species were identified, namely *Nigrogranacamelliae*, *N.guttulata*, *N.longiorostiolata* and *N.neriicola*, Additionally, our known species, namely *N.acericola*, *N.magnoliae*, *N.oleae* and *N.thymi*, were reported from medical plants as new host records. These isolates were associated with terrestrial habitat and collected from medicinal plants in nine plant families, including Apocynaceae, Berberidaceae, Buxaceae, Celastraceae, Eucommiaceae, Fabaceae, Primulaceae, Rutaceae, and Theaceae.

Species within the genus *Nigrograna* exhibit considerable morphological similarity, often complicating species delimitation based solely on morphological traits (Jaklitsch and Voglmayr 2016; Zhang et al. 2020; Lu et al. 2022; Li et al. 2023). As such, molecular data play a critical role in species identification. For example, Jaklitsch and Voglmayr (2016) demonstrated that morphologically similar species, such as *N.coffeae* and *N.camelliae*, can be distinguished phylogenetically. These species sequence divergence across multiple loci, including (15/514 bp, 2.9%, 1 gap), LSU (11/698 bp, 1.6%, without gaps), *rpb2* (74/739 bp, 10.0%, without gaps) and *tef1-α* (28/914 bp, 3.1%, without gaps), highlighting the importance of molecular analysis for accurate taxonomic placement. *Nigrograna* is a worldwide distributed genus, with species reported from Asia (Dayarathne et al. 2020; Mapook et al. 2020; Zhang et al. 2020; Lu et al. 2022; Li et al. 2023), the Americas, and Europe (Jaklitsch and Voglmayr 2016; Hyde et al. 2017; Kolařík et al. 2017; Tibpromma et al. 2017; Kolařík 2018; Zhao et al. 2018; Dayarathne et al. 2020; Wanasinghe et al. 2020). While certain species, such as *N.carollii*, *N.peruviensis* and *N.yasuniana*, have been reported as endophytes on various hosts (Kolařík et al. 2017), the majority of known species are saprotrophs on the bark or corticated twigs and branches of various hardwoods (Jaklitsch and Voglmayr 2016; Mapook et al. 2020; Zhang et al. 2020; Lu et al. 2022; Hu et al. 2023; Li et al. 2023). Reports of *Nigrograna* species on flowers, fruits, leaves, or herbaceous plants are rare, indicating a preference for woody hosts. Consistent with these findings, the isolates in this study were primarily recovered from the branches of medicinal woody plants, such as *Eucommiaulmoides* (Eucommiaceae), *Gymnosporiaacuminata* (Celastraceae), and *Mahoniabealei* (Berberidaceae).

It is noteworthy that *Nigrogranamagnoliae* was isolated from the bark of *Eucommiaulmoides*, which is a primary medicinal component of the plant. The quality of medicinal plants is closely tied to their clinical efficacy, and the presence of fungal species such as *N.magnoliae* raises important questions about the potential impact of fungal colonization on the medicinal properties of their hosts (Balekundri and Mannur 2020; Rasool et al. 2020; Ali et al. 2021). This finding warrants further investigation to assess whether *N.magnoliae* could affect the quality or bioactive compounds of *E.ulmoides*. In addition to their ecological diversity, certain species within *Nigrograna* have been found to produce bioactive secondary metabolites. For instance, *Nigrogranarubescens* has been reported to produce naphthoquinone compounds, which are known for their broad spectrum of biological activities (Naysmith et al. 2017; Mack et al. 2024). These metabolites share structural similarities with those found in *N.antibiotica*, which also produces bioactive compounds (Stodůlková et al. 2014). Such findings suggest that members of *Nigrograna* have significant potential for biotechnological applications, particularly in drug discovery. Understanding the relationships between *Nigrograna* species and their medicinal plant hosts, as well as the impact of fungal colonization on the quality of these plants, remains a critical area of research.

In conclusion, this study highlights the diversity of *Nigrograna* species associated with medicinal plants in Southwest China and underscores the importance of integrating morphological and molecular data for accurate species identification. Given the potential ecological and economic implications of *Nigrograna* colonization on medicinal plants, continued research is essential. Detailed taxonomic and ecological studies of *Nigrograna* from medicinal plants will provide valuable insights into the species diversity, host specificity, and potential biotechnological applications of this genus. Ongoing efforts to collect and analyze fresh isolates will further enhance our understanding of the genus and its broader ecological and medicinal significance.

## Supplementary Material

XML Treatment for Nigrograna magnoliae

XML Treatment for Nigrograna longiorostiolata

XML Treatment for Nigrograna acericola

XML Treatment for Nigrograna camelliae

XML Treatment for Nigrograna oleae

XML Treatment for Nigrograna thymi

XML Treatment for Nigrograna neriicola

XML Treatment for Nigrograna guttulata

## ﻿Additional information

### Author ORCIDs

Hong-Zhi Du https://orcid.org/0000-0003-0350-4530

Yu-Hang Lu https://orcid.org/0009-0005-9819-3182

Ratchadawan Cheewangkoon https://orcid.org/0000-0001-8576-3696

Jian-Kui Liu https://orcid.org/0000-0002-9232-228X

### Data availability

All of the data that support the findings of this study are available in the main text.
